# Traffic lights detection and tracking for HD map creation

**DOI:** 10.3389/frobt.2023.1065394

**Published:** 2023-03-03

**Authors:** Simone Mentasti, Yusuf Can Simsek, Matteo Matteucci

**Affiliations:** Politecnico di Milano, DEIB, Milan, Italy

**Keywords:** HD-map, traffic-lights, detection, mapping, tracking

## Abstract

HD-maps are one of the core components of the self-driving pipeline. Despite the effort of many companies to develop a completely independent vehicle, many state-of-the-art solutions rely on high-definition maps of the environment for localization and navigation. Nevertheless, the creation process of such maps can be complex and error-prone or expensive if performed *via* ad-hoc surveys. For this reason, robust automated solutions are required. One fundamental component of an high-definition map is traffic lights. In particular, traffic light detection has been a well-known problem in the autonomous driving field. Still, the focus has always been on the light state, not the features (i.e., shape, orientation, pictogram). This work presents a pipeline for lights HD-map creation designed to provide accurate georeferenced position and description of all traffic lights seen by a camera mounted on a surveying vehicle. Our algorithm considers consecutive detection of the same light and uses Kalman filtering techniques to provide each target’s smoother and more precise position. Our pipeline has been validated for the detection and mapping task using the state-of-the-art dataset DriveU Traffic Light Dataset. The results show that our model is robust even with noisy GPS data. Moreover, for the detection task, we highlight how our model can correctly identify even far-away targets which are not labeled in the original dataset.

## 1 Introduction

In the last decade, interest in autonomous driving has rapidly grown inside research institutes and industries. As a result, the desire to build autonomous vehicles without an actual driver is getting real. Many notable attempts have been made to fulfill this goal, and several significant milestones have been reached. In addition, there have been attempts to operate autonomous vehicles on public streets. However, these efforts have been restricted to a subset of the full driving task, e.g., highway-only, parking-only, or throttle/brake only, as reported in Levinson et al. (2011), Yurtsever et al. (2020) and Cho et al. (2021).

To accomplish all autonomous driving tasks, computer systems should carefully reproduce the human ability to perceive the environment and behave accordingly. In particular, autonomous terrestrial vehicles used in urban areas must perceive the environment as precisely as possible. Therefore, many of the most advanced driver-assistance systems (ADAS) and automated driving systems (ADS) rely on some form of high-definition (HD) maps. Indeed, while many manufacturers are working on a fully autonomous vehicle system, able to navigate the environment without using pre-built knowledge of the driving scenario, many of the advanced solutions running in urban environments relay some HD Map, as shown in Bock (2021). This happens mostly because, as shown by Liu et al. (2020) the usage of HD Map is highly beneficial for the navigation process and can significantly increase the localization accuracy of the vehicle. These maps impact the overall system confidence, reduce system computational resource needs, help to improve comfort and convenience, and ultimately contribute to system safety thanks to their centimeter-level accuracy. HD maps are often captured using an array of sensors, such as LiDARs, radars, digital cameras, and GPS, as depicted in Karls and Mueck (2018); alternatively, they can also be constructed using aerial imagery, as proposed by Javanmardi et al. (2017).

HD-maps usually include elements such as buildings, barriers, road shapes, road markings, curbs, traffic signs, and traffic lights, as described by Zang et al. (2018). Moreover, they are helpful for 3D object detectors since they provide strong priors that can boost performance and robustness. For instance, HDNET by Yang et al. (2018) takes advantage of these priors to extract geometric and semantic features from the HD maps to be used in 3D object detection. Indeed, thanks to HD maps, unlabeled driving logs can be self-annotated. Furthermore, in Wilson et al. (2020), authors backproject the 2D instance segmentation on the image plane into 3D cuboids in LiDAR space using the lane geometry present in Argoverse’s HD maps by Chang et al. (2019) to constrain otherwise ambiguous orientations. Other use cases for the HD maps are in sensor validation and in the testing of ADS functions in simulation as they provide accurate information, which can be considered as a ground-truth, as done by Altec (2022).

However, creating HD maps requires a lot of human effort, which is error-prone and inefficient. Maps are built using 3D point clouds and relevant semantic information; therefore, human resources are required to label the latter, such as the position of the lanes, roads, traffic signs, and traffic lights. An obvious shortcoming of this method is the lack of accuracy; also, labeling point clouds is known to be very tedious and can significantly slow down the HD maps building process, as highlighted in Elhousni et al. (2020). Automating such manual work is critical to improve the efficiency of the process and the quality of HD Maps. To achieve this automation, different approaches have been employed. Heuristics based, like (Yang et al., 2013; Ordóñez et al., 2017; Yan et al., 2017). Recently, also deep learning approaches on 3D point clouds have been used for such automation, as shown in Jiao (2018). In this case, the most common solutions are based on Pointnet by Qi et al. (2017a), and its variants, like (Qi et al., 2017b) and (Zhou and Tuzel, 2018). In Elhousni et al. (2020), authors propose a novel method capable of generating labeled HD maps from raw sensor data, which uses a deep learning approach. In the paper, they present road mapping and lane mapping modules and combine them to have driveable areas and lanes for HD map creation. However, there is no contribution for the stationary map layer, consisting of traffic lights, road/traffic signs, or building labels.

Although in literature there are studies on the detection of light poles and pole-like objects, like (Yu et al., 2014), and (Yan et al., 2017), only a few studies exist on traffic light detection for map creation; a good example is the one presented in Fairfield and Urmson (2011). Indeed, traffic light detection is widely addressed in the literature, but there are still some challenges to be faced. In particular, recognition in adverse conditions like rain or snow, early recognition (detecting traffic lights at greater distances), and recognition under different illumination settings, as illustrated by Possatti et al. (2019). Traffic light detection also shares the issues of small object detection, especially when early recognition is considered. For instance, in Bosch Small Traffic Lights Dataset by Behrendt and Novak (2017) the different traffic light sizes within the training set vary between approximately 1 and 85 pixels in width, with the mean of 11.3 pixels. Similarly, the DriveU Traffic Light Dataset (DTLD) by Fregin et al. (2018a) contains traffic lights with a width of 1–88 pixels and a mean of 14.67 pixels. Many studies have focused on traffic light detection, and state recognition, like (Behrendt et al., 2017), and (Possatti et al., 2019), or (de Mello et al., 2021). These studies perform well and detect tiny traffic light objects in the input images. However, for HD map creation, the physical information of the traffic lights, such as bulb numbers, directions, and pictograms, is also relevant. At the same time, the state is not so important since it is variable. Therefore, detecting these attributes requires a multi-label single-class approach that separates traffic light detection for HD map creation from the well-known traffic light detection and recognition task.

**FIGURE 1:**
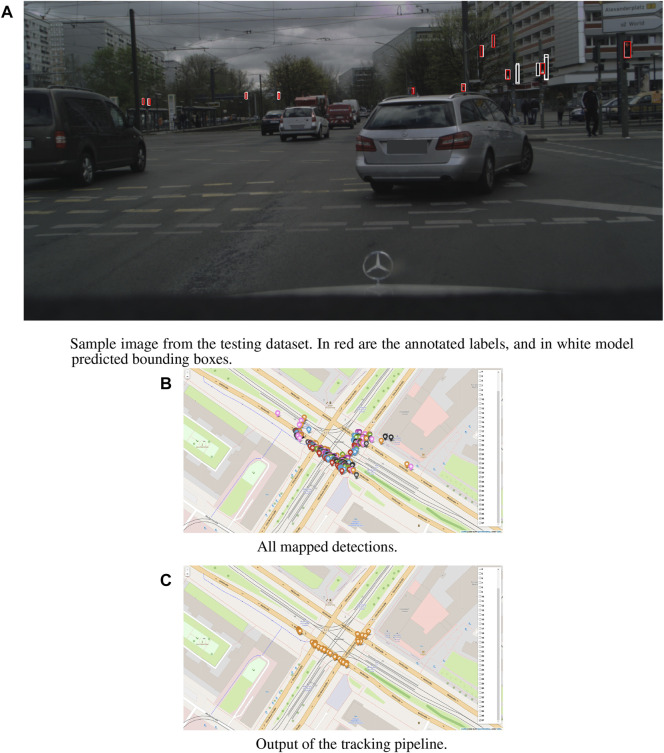
Core components of the traffic lights-HD map creation pipeline. The output of the network with lights positioned on the image plane **(A)**. Georeferenced position of the lights **(B)**. And the tracked position of the lights **(C)**.

**FIGURE 2:**
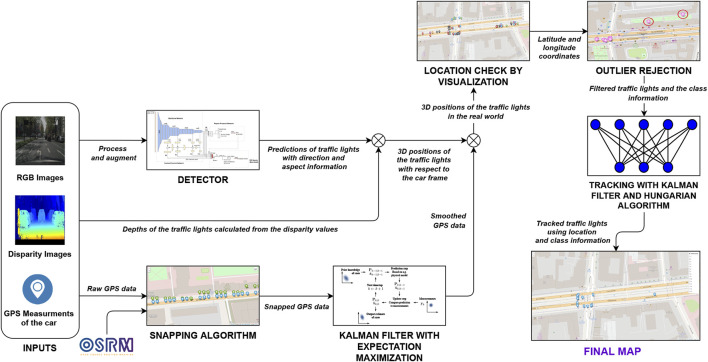
Schema of our pipeline for traffic lights map creation. The input of the system are RGB images, disparity maps and GPS positions of the vehicle. The output is a map with the GPS position of all detected lights.

This work focuses on traffic light detection and tracking using deep learning methods for HD map creation. The core components of the system, the light detector, and the resulting traffic lights map are depicted in Figure 1. Our pipeline, shown in Figure 2 takes as input an image and a disparity map computed *via* stereovision, plus the vehicle position acquired using a simple GPS sensor with 2*m* CEP (Circular error probable) error. The final output is a georeferenced map of fully described lights, with shape, orientation, and type information. The core contributions of this work can be summarized as:

• development of a single-label object detector for traffic light state detection.
• development of a multi-label object detector for traffic light features detection.
• development of a mapping pipeline that, using data from a stereo camera and GPS, can compute an accurate map of fully characterized traffic lights.

The paper is structured as follows; in Section 2 we present a state-of-the-art overview of traffic light detection, focusing on available datasets and deep learning models employed for this task. Then, in Section 3.1 we present the different models developed for the light detection task, first using single-label detectors and then multi-label ones. Next, in Section 3.2 we present the pipeline designed to convert the detection on the image plane to a 2D georeferenced map of the lights. Finally, in Section 4 we compare the performance of the different trained models, and we display the results of the map creation algorithm.

## 2 Related works

Thanks to the growing interest in autonomous driving in the last 10 years, various traffic light datasets have been released to train deep learning models. Nevertheless, as previously stated, the focus is, in most cases, only on the state of the light and not the shape and features. Nowadays, it is possible to find five large datasets for this task. One of the first datasets for traffic light detection is the LaRA Traffic Lights Recognition Dataset (De Charette and Nashashibi, 2009a). It contains 11,179 frames recorded in 8 min 49 s at 25 FPS in Paris. Frames are in 8bit RGB format with 640 × 480 resolution. The camera is mounted behind the interior rear-view mirror, and the vehicle speed is lower than 50 *km*/*h* during recording. There are 9,168 instances of unique traffic lights which are hand-labeled. The labels are only green, orange, red, and ambiguous. The Bosch Small Traffic Lights Dataset (BSTLD) (Behrendt and Novak, 2017) is an accurate vision-based traffic light detection dataset. The scenes from the dataset cover a decent variety of road scenes and typical difficulties such as busy street scenes, suburban multilane roads with varying traffic density, dense stop-and-go traffic, and many others. The dataset contains 13,427 camera images at 1280 × 720 pixels and has about 24,000 annotated traffic lights. The annotations include bounding boxes of traffic lights and each traffic light’s current state (active light). The LISA Traffic Light Dataset (Philipsen et al., 2015) is collected based on footage from San Diego, California, USA roads to provide a common basis for comparison of traffic light recognition research. The dataset consists of continuous test and training video sequences that last 23 min and 25 s, 43,007 frames, and 113,888 annotated traffic lights. The serieses are captured by a stereo camera mounted on the roof of a vehicle driving during both night and daytime with varying light and weather conditions. The resolution of the images is 1280 × 960, and the field of view (FoV) is 66°. BDD100K (Yu et al., 2020) is constructed as a driving dataset for heterogeneous multitask learning but includes traffic light labels. It is a diverse and large-scale dataset of visual driving scenes developed for multiple tasks (e.g., lane detection, drivable area segmentation, road object detection, etc.). The dataset consists of over 100K diverse video clips, which are 40 s long and collected from more than 50K rides covering New York, San Francisco Bay Area, and other regions in different weather conditions. One of the latest and most extensive datasets for traffic light detections, and the one employed in this work, is the DriveU Traffic Light Dataset (DTLD) (Fregin et al., 2018b), which contains 292,245 annotations from 2048 × 1024 images. It has the highest number of annotations and the highest resolution compared to other datasets. The images are captured in 10 cities in Germany at a frame rate of 15 Hz. Differently from the other datasets, the labels have detailed descriptions such as direction, aspects, orientation, state, pictogram, occlusion, relevancy, and reflection. For this reason, we decided to use this specific dataset since it was the only one that provided all the required information for precisely mapping the lights. A comparison of the presented traffic light detection datasets is given in Table 1.

**TABLE 1:** Comparison of the statistics of the TLD datasets.

	LARA	LISA	BSTLD	BDD100K	DriveU (v2.0)
Resolution [WxH]	640 × 480	1280 × 960	1280 × 720	1280 × 720	2048 × 1024
Depth [bit]	8	8	8, 12	8, 16	8, 16
Frame Rate [Hz]	25	16	−	30	15
Annotations	9,168	113,888	24,242	265,906	292,245
Cities	1	1	1	4+	10
Disparity data	−	−	−	−	+
Classes	S	S	S, P	S, O	S, P, D, A, O, R, RF, OR
Night Images	−	+	−	−	−

Classes: S, States available; P, pictogram available; O, Occlusion available; A, aspects available; R, Relevancy available; D, direction with respect to vehicle available; RF, Reflection available; OR, installation orientation available.

An extensive overview of approaches to traffic light detection (TLD) and classification up to 2016 is given in Jensen et al. (2016). In literature, the TLD problem can be categorized into model-based methods and learning-based methods. The firsts are currently the most widely used. They rely on heuristically determined models using shape, color, and intensity and make strong assumptions about the location and size of traffic lights. Many previous works in this category have used the color thresholding method for hypotheses generation, like in Omachi and Omachi (2009). Others use the circular shape of traffic lights as a feature for detection through the Hough transform (Omachi and Omachi, 2009) or bulb detection (De Charette and Nashashibi, 2009b). Further, prior map information is utilized to create detection hypotheses and increase the detection rate (Possatti et al., 2019). On the other hand, the learning-based model has escalated in the last years and performs better day by day with the development of the deep learning algorithms and the improvement of the dataset quality (Fernández et al., 2018).

For example, in Bach et al. (2018), DTLD is used for training a Faster R-CNN model (Ren et al., 2015), with ResNet50 backbone, leveraging on pretrained weights on ImageNet (Deng et al., 2009). The authors also implement a YOLOv3 anchor generation approach (Redmon and Farhadi, 2018) for their model. In Possatti et al. (2019), YOLOv3 is trained from scratch on DTLD, and the detector is combined with prior maps to recognize relevant traffic lights. Similarly, authors of (Müller and Dietmayer, 2018) adapt a single shot detection (SSD) approach by having structural changes on the default SSD (Liu et al., 2016) to detect objects smaller than ten pixels without increasing the image size. In the work, the authors have trained a model with Inception backbone (Szegedy et al., 2015) and present an extensive evaluation on DTLD. In de Mello et al. (2021), the images are replicated to cope with the class imbalance in DTLD, and a total of 70k images are obtained for training the Faster RCNN with ResNet101 backbone. They also provide an evaluation on many other datasets, including Udacity, LISA and LaRA. A different approach is to model the temporal context for TLD using RNNs as shown in Björnsson and Westerberg (2021). As a detector, YOLOv4 (Bochkovskiy et al., 2020) is also trained on DTLD, and its augmentation techniques are implemented to further increase the variance of the data.

In order to fill in occasional gaps from the detector, researchers employ trackers in many different field. Visual object trackers use a similarity measurement metric on the image to determine the target position in a neighborhood around the position in the previous image frame. Multiple metrics exists based on pixel-wise similarity, histogram similarity (Comaniciu et al., 2003), HoGs, feature points, lines, and neural network encoders (Hong et al., 2015). For the specific TLD task, it is also possible to implement a fictitious motion model to estimate the relative movement of the traffic lights to the moving vehicle in the image frames to track the positions. In Behrendt et al. (2017), authors use an odometry-based motion model of the ego-vehicle to predict the movement and a neural network to improve the tracked positions. The neural network receives an image and a crop from a candidate position, then predicts the candidate’s offset (*u*, *v*) from the tracked object and an error estimate that represents the uncertainty of the position estimate, which is used to trigger the update of the prototype image. The use of Hidden Markov Models is proposed in Gomez et al. (2014) to filter errors in state estimation, which increases the detection rate. In Trehard et al. (2014), the authors propose an Interacting Multiple Model filter to track the position and the status of traffic lights.

Compared to the presented state of the art, in this work, we extend the most recent object detection models adapting them to work on DTLD in a multi-label manner. We then perform a comparative analysis of these models to identify the best-performing one, in terms of accuracy and computational requirements. Finally, using detections, GPS measurements, and disparity images, the 3D positions of each traffic light are estimated, and a Kalman Filter is implemented to smoothen the vehicle data and perform tracking. With this information, we build a map of fully characterized traffic lights in the surveying area.

## 3 Methods

In this section, we present our pipeline for traffic light detection and mapping. We first analyze the problem of light detection for single and multi-label tasks. Then, using the output of this first stage, we illustrate the pipeline of map creation.

### 3.1 Traffic light detection

In this section, we propose methods for unified traffic light detection and classification using different deep learning detection models. At first, we present the single label multi-class problem, which is the common task for TLD with state recognition. Afterward, we extend the results of the single label problem and focus on multi-label single class which is more relevant to HD map creation. Indeed, beside the traffic light state, physical information such as direction, bulb numbers, and the pictogram of the traffic lights are essential for HD maps. We discuss these two different tasks in Section 3.1.1 and Section 3.1.2 respectively.

#### 3.1.1 Single-label detector

In the DTLD dataset, we have five states for traffic lights: off, red, yellow, red-yellow, and green, which are the classes to recognize in a single label task. There are also many traffic lights with unknown states: these could be occluded, directed such that the bulbs are not observable, reflected, and far away to label the state correctly. Therefore, in this task we have filtered out unknown state labels. In this work, we have trained and compared the models from Scaled-YOLOv4 (Wang et al., 2021a) and YOLOR (Wang et al., 2021b). Previously, the authors have shown that YOLOv4 (Bochkovskiy et al., 2020), based on the CSP (Wang et al., 2020) approach, scales both up and down and modifies the networks’ depth, width, and resolution while maintaining optimal speed and accuracy. For Scaled-YOLOv4, they also have scaled the network structure, which results in state-of-the-art results for the MS COCO dataset (Lin et al., 2014). These two network families have been chosen mainly due to inference/training time while considering the best accuracy according to the literature.

YOLOv4-p7 is the largest and best-performing model in the first family (Wang et al., 2021a); however, training on DTLD takes quite a long time and computational effort. Its default input size is 1536 × 1536, which requires at least two GTX 1080 Ti GPUs to train with a batch size of 1. On the other hand, the smallest model is YOLOv4-tiny, which is developed for low-end devices, and therefore scarifies too much in terms of accuracy to run on limited resources. YOLOv4-CSP provides a good trade-off between the performance and the computational effort. From the second family, YOLOR-d6 has the best results on COCO, which is comparable to YOLOv4-p7. In addition, it is much faster during inference, as stated in by Wang et al. (2021b).

We have trained YOLOv4-CSP with different configurations. For the first setup, we filtered out all labels smaller than 10 pixels to check the model’s performance on a setting that does not include tiny objects. This makes it possible to evaluate the fitness of the MS COCO pre-trained weights to DTLD and to determine if these weights are feasible to use or if training from scratch is better. Before training, the model analyzes the labels to evolve the default anchors with a genetic algorithm by using k-means for the nine best anchors, and these are used during training. Indeed, generated anchors have a better match with ground-truths. For this configuration, the batch size is 14. The input resolution is 640 × 640, the default size of the model trained on MS COCO. Stochastic Gradient Descent (SGD) optimizer is used with an initial learning rate of 0.01, a momentum of 0.937, and a weight decay rate of 0.0005. Training IoU threshold is set to 0.2, which decides the objectness. We did not add specific augmentation techniques, leaving it to the default ones; also, the Mosaic technique is used. For this problem we decided not to use Multi-scale, rectangular training and synchronized batch normalization.

In the second setup, the model has been trained using all the labels, with the same input size and augmentations as the first setup. Finally, as the last configuration for this model, we have changed the input size to the original size of the images in DTLD, 2048 × 1024, and trained the model with newly generated anchors. The goal is to enlarge the small labels in the other two setups and let the model see more labels to train, which is effective on the detection performance.

Moreover, YOLOR-d6 has been trained with all labels and 1280 × 1280 input size, which is the default setting for this model. For this model, we employed the same augmentation techniques and custom anchor generator as the first one. Multi-scale training and synchronized batch normalization (sync-bn) are not used, but rectangular training is active. The network uses SGD optimizer since it has the same settings as the first model.

#### 3.1.2 Multi-label detector

For the multi-label training, the classes we have used refer to two subcategories of the labels, bulb number, and traffic light direction. For the first one, the dataset considers one aspect, two aspects, and three aspects of lights, which are the classes available for bulb number. For the direction, we have all four possible orientations of the traffic light. Thus, there are seven classes in the class list, and each traffic light has two labels from this list. Considering the label distribution in the dataset, we have filtered out all elements which do not present these features; lights that are horizontal, occluded, reflected, and four aspects of traffic lights are removed from training and validation sets.

At first, the models have been trained on 10% of the train-set and validated with the same portion, keeping label distribution the same as the entire dataset. According to the precision, recall, mAP results, and training time of the models with different settings such as resolution and augmentation techniques, we have decided to refine the training of YOLOv4-CSP and YOLOR-d6 models on the full dataset. The two models trained are one-stage detectors, and we have decided to compare their performances to a two-stage detector since the detection task here includes very small objects.

The first model, YOLOv4-CSP-multi, has the same configuration as CSP640. Mosaic data augmentation, color space augmentation, random flip, and class label smoothing are the augmentations used. The model is trained for 100 epochs, with a total batch size of 24.

The second model, YOLOR-D6-multi, has the same settings as YOLOR-D6. Additionally, the input size is 1024 × 1024 because the results are similar to the 1280 × 1280 resolution, but the time difference is substantial for the 10% dataset. The augmentations are the same with YOLOv4-CSP-multi. The total batch size is 4, and the model is trained for 50 epochs. The difference between the epochs of YOLOv4-CSP-multi and YOLOR-D6multi is due to the results on the 10% dataset. The results show that the latter achieves a similar performance to the first one in around half of the epochs. On the other hand, it lasts longer to train YOLOR-D6multi due to model size and bigger input resolution. Thus, it is interesting to compare the results of the two models.

The well-known Detectron2 (Wu et al., 2019) framework by Facebook AI Research contains several two-stage detectors with different backbones evaluated on the COCO Object Detection, Instance Segmentation, Person Keypoint Detection, and also other datasets such as LVIS (Gupta et al., 2019), Cityscapes (Cordts et al., 2016) and Pascal VOC (Everingham et al., 2010). Moreover, ablations for deformable convolution (Dai et al., 2017), Cascade R-CNN (Cai and Vasconcelos, 2018), and different normalization methods are available too. We have chosen Faster R-CNN object detection network with ResNet50 and Feature Pyramid Network (Lin et al., 2017) as the backbone (Faster R-CNN-R50-FPN). In addition, we have also trained Faster R-CNN with ResNeXt-101-32 × 8d (Faster R-CNN X-101) backbone, which is the best performing model on COCO Object Detection, to compare the performance on DTLD.

The Detectron2 framework does not support multi-label detection, and the whole pipeline is constructed according to single label (and single/multi-class) detection. Therefore, we have extended the original architecture to the multi-label classification problem and made the model learn multi-label detections. We have created a new dataset mapper by inheriting the default one to keep the annotations in the validation process and control the augmentations for training and testing directly from the mapper. We have changed the function that reads the images as we have done for other models. In addition, a function that creates a dataset dictionary required by the pipeline has been made. To evaluate the model and compare its performance to other models, the same evaluation procedure has been adapted to Detectron2, which cannot evaluate multi-label tasks by default. Moreover, we have added a checkpoint class to save the best-performing model in the AP50 metric during training instead of just saving the last model.

In addition to the changes mentioned, we have also modified the models for our task. Box regression loss calculation has been altered to have multi-label ground truths as input and determine foreground indices by using them. Instead of cross-entropy loss with softmax, we have used binary cross-entropy with logits, one per class. One-hot encoded ground truths have been replaced with multi-hot vectors. Since the model outputs logits, the prediction probabilities have been calculated by sigmoid. Lastly, we have adopted a custom anchor generator that uses k-means (Hamerly and Elkan, 2003) and IoU metrics (Rezatofighi et al., 2019) to calculate the best fitting anchors for labels in DTLD, to replace the default anchors designed for the COCO dataset. For inference, the function that returns a list of tensors of predicted class probabilities for each image calculates the probabilities with softmax, which is not appropriate for multi-label tasks. The changed version uses instead a sigmoid.

### 3.2 Map creation

The output of the detectors is a list of 2D bounding boxes with features on the image plane. To construct a map of the lights’ position further processing is required. The first additional information required is the vehicle position when the image is acquired. This is retrieved using a commercial GPS sensor, with CEP of 2*m*. This means that these measurements are not accurate enough to be directly used as a single localization source. In addition, many of them do not correspond to a point in any road segment due to multipath and GPS error. To cope with this, we have filtered the erroneous measurements outside of the local area coordinates, measurements with errors in position greater than 20*m*. Then, since we can assume the vehicle is driving on a road we employed a matching service to snap the measures to the road segments by utilizing OSRM nearest service (Luxen and Vetter, 2011). To further improve the accuracy of the GPS data and track the vehicle position, we have also implemented Kalman Filters with Expectation Maximization algorithm (Moon, 1996). The GPS data are converted from latitude and longitude to local ENU coordinates, then the filter is fed with the vehicle position and the speed data. The state Equation 1 and measurement Equation 2 for each sequence are:

$${\tilde{x}}_{t}=F{\tilde{x}}_{t-1}+Gu_{t}+w_{t}$$ (1)

$$y_{t}=H{\tilde{x}}_{t}+v_{t}$$ (2)

where *F* is the state transition matrix, *G* is the control matrix, *H* is the observation matrix, *u*_*t*_ is the control input, $w_{t}∼N\left(0,Q_{n}\right)$ is process noise, and $v_{t}∼N\left(0,R_{n}\right)$ is measurement noise. The system state is defined as:

$$\tilde{x}={\left[\begin{array}{c} x,\dot{x},y,\dot{y},\theta ,\dot{\theta } \end{array}\right]}^{T}$$ (3)

While the two matrices F and H are:

$$F=\left[\begin{array}{cccccc} 1 & \Delta t & 0 & 0 & 0 & 0\\ 0 & 1 & 0 & 0 & 0 & 0\\ 0 & 0 & 1 & \Delta t & 0 & 0\\ 0 & 0 & 0 & 1 & 0 & 0\\ 0 & 0 & 0 & 0 & 1 & \Delta t\\ 0 & 0 & 0 & 0 & 0 & 1 \end{array}\right]$$ (4)

$$H=\left[\begin{array}{cccccc} 1 & 0 & 0 & 0 & 0 & 0\\ 0 & 1 & 0 & 0 & 0 & 0\\ 0 & 0 & 1 & 0 & 0 & 0\\ 0 & 0 & 0 & 1 & 0 & 0\\ 0 & 0 & 0 & 0 & 1 & 0\\ 0 & 0 & 0 & 0 & 0 & 1 \end{array}\right]$$

We defined our state vector as ${\tilde{x}}_{t}$ , where *x* and *y* are the Easting and Northing values obtained from the latitude and longitude GPS measurements respectively, and *θ* is the heading angle of the car. $\dot{x}$ and $\dot{y}$ are the velocities projected in the East and North directions from the velocity measurement of the car, and ${\dot{\theta }}_{n}$ is the yaw rate measurement. *F* and *H* matrices are constructed according to the states and measurements (4). The state prediction (5) and state update (6) equations are as follows:

$${\hat{x}}_{t+1|t}=F{\hat{x}}_{t|t}$$ (5)

$${\hat{x}}_{t+1|t+1}={\hat{x}}_{t+1|t}+K_{t+1}\left(y_{t+1}-H{\hat{x}}_{t+1|t}\right)$$ (6)

The output of the filter is a more accurate localization of the car. To reconstruct a map, the last required information is the relative position of each light to the vehicle. This can be computed by fusing the detection on the image plane with the depth calculated from stereovision provided by the dataset. By calculating the mean distance of all the points in a detected bounding box, it is possible to get an estimate of the light’s relative distance to the camera. Since the disparity images are still subject to noise, some outliers are present in the projections. Therefore, each traffic light with a distance greater than 150*m* is removed from the detections. Exploiting the extrinsic camera calibration parameters and the vehicle’s state information from the Kalman Filter, combined with the depth, it is possible to compute the absolute position of each detection.

Lastly, we have implemented a multi-object tracking algorithm consisting of Kalman Filters and Hungarian Algorithm (Kuhn, 1955) to refine the light’s position with consecutive detections. We have applied filters to each traffic light and made the assignments using the Hungarian Algorithm. Since the objects we are tracking are all static, the Kalman Filters do not perform prediction but only update the states and covariances. As a cost for the assignment process, to perform intra-frame association, we have used Euclidean distance if the detected class information of the traffic light corresponds to any of the tracks; otherwise, we set a high cost to eliminate class mismatches. Furthermore, we delete the tracks which have a higher number of skipped frames than a fixed threshold, or are outside the camera field of view.

## 4 Experimental results

We have evaluated the detectors presented in Section 3.1 on the DTLD test set that contains 12.453 images with 72.137 traffic light annotations with a very similar distribution to the train set. We have included all the labels in the test set. In Table 2, class-based evaluation results of the single-label models with an NMS IOU threshold of 0.5 and a confidence score threshold of 0.4 are given. In addition, the number of annotations for the classes and training resolutions of the models are available. We tested the models with a resolution of 2048 × 1024, i.e., the original size.

**TABLE 2:** Class-based comparison of the models for the singe-label task.

Models	(1)	(2)	(3)	(4)	(1)	(2)	(3)	(4)	(1)	(2)	(3)	(4)
Classes	All (72137)				Off (18696)				Red (20790)			
mAP@0.5	0.655	0.641	**0.717**	0.714	0.26	0.246	0.363	**0.381**	0.783	0.754	**0.833**	0.812
mAP@0.5:0.95	0.369	0.347	0.487	**0.488**	0.112	0.089	0.247	0.200	0.436	0.397	0.546	**0.563**
Precision	0.72	0.704	**0.809**	0.799	0.469	0.426	**0.717**	0.656	0.859	0.772	0.820	**0.895**
Recall	0.551	0.545	0.572	**0.585**	0.0826	0.116	0.009	0.139	0.649	0.682	0.761	**0.684**
Classes	Yellow (3033)				Red-yellow (950)				Green (29618)			
mAP@0.5	0.726	0.723	0.765	**0.779**	0.654	0.631	**0.731**	**0.731**	0.851	0.852	**0.893**	0.868
mAP@0.5:0.95	0.424	0.408	0.532	**0.545**	0.389	0.377	0.514	**0.522**	0.485	0.463	0.597	**0.610**
Precision	0.714	0.778	**0.793**	0.765	0.692	0.714	**0.822**	0.760	0.868	0.832	0.895	**0.917**
Recall	0.619	0.588	0.648	**0.699**	0.623	0.531	0.592	0.614	0.779	0.811	**0.851**	0.790

Models: (1): YOLOv4-CSP 640 × 640 trained with 10 pixels limit. (2): YOLOv4-CSP 640 × 640 trained with all labels. (3): YOLOv4-CSP 2048 × 1024 trained with all labels. (4): YOLOR-D6 1280 × 1280 trained with all labels.

Bold values indicate the best results in each row and column.

YOLOv4-CSP trained with 2048 × 1024 resolution and YOLOR-D6 trained with 1280 × 1280 performs quite close to each other and outperforms the other models, but the margin is not very large. All the models have acceptable results, especially for the traffic lights annotated as red and green which are more than half of the annotations. Surprisingly, YOLOv4-CSP trained with labels bigger than 10 pixels in width shows satisfactory performance, particularly when the smaller number of training labels and the input resolution is considered. Although there are plenty of labels smaller than 5 pixels in the original size images, the models cannot learn fine semantic information since the spatial information is coarse when the input resolution is reduced to the one required by the network, i.e., 640 × 640.

For the multi-label task, we have evaluated three models using the updated labels of the DTLD, which contains more than 70.000 annotations even after the filtering process discussed in Section 3.1.2. We have tested the models with a resolution of 2048 × 1024. According to the metrics, the largest model, YOLOR-D6, performs best, and Faster R-CNN-R50-FPN shows similar performance. YOLOv4-CSP cannot reach the same level of performance. Although we have trained it for 2x epochs, the input resolution worsens the predictions. Table 3 provides a quantitative analysis of the results and inference details. The model were evaluated with the best performing setup. In particular, the two Yolo version have the same NMS and confidence threshold value, while Faster R-CNN have different values due to the structure of the network, which performed best with 0.8 confidence threshold.

**TABLE 3:** Class-based comparison of the models for the multi-label task.

Models	1*	2*	3*	1*	2*	3*	1*	2*	3*	1*	2*	3*
Classes	One Bulb			Two Bulbs			Front Directed			Front Directed		
mAP@0.5	0.397	**0.452**	0.409	0.465	**0.550**	**0.540**	0.610	0.779	**0.785**	0.581	0.815	**0.818**
mAP@0.5:0.95	0.196	**0.263**	0.229	0.233	**0.321**	0.281	0.362	**0.543**	0.500	0.333	**0.557**	0.374
Precision	**0.752**	0.734	0.703	**0.837**	0.796	0.680	**0.954**	0.939	0.850	**0.940**	0.923	0.854
Recall	0.046	**0.175**	0.127	0.102	0.319	**0.399**	0.269	0.609	**0.665**	0.224	0.694	**0.740**
Classes	Back Directed			Right Directed			Left Directed			Weighted All		
mAP@0.5	0.000	**0.269**	0.105	**0.500**	0.320	0.203	**0.500**	0.304	0.365	0.559	**0.701**	0.686
mAP@0.5:0.95	0.000	0.187	0.045	0.150	**0.168**	0.078	0.100	**0.165**	0.016	0.302	**0.471**	0.370
Precision	0.000	**0.494**	0.179	**1.000**	0.615	0.376	**1.000**	0.572	0.189	**0.925**	0.868	0.766
Recall	0.000	0.061	**0.107**	0.000	0.032	0.049	0.000	0.055	0.096	0.195	0.528	**0.574**

Models: 1*: YOLOv4-CSP 640 × 640 tested with 0.5 NMS and 0.4 confidence threshold. 2*: YOLOR-D6 1280 × 1280 tested with 0.5 NMS and 0.4 confidence threshold. 3*: Faster R-CNN-R50-FPN 2048 × 1024 tested with 0.0 NMS and 0.8 confidence threshold.

**FIGURE 3:**
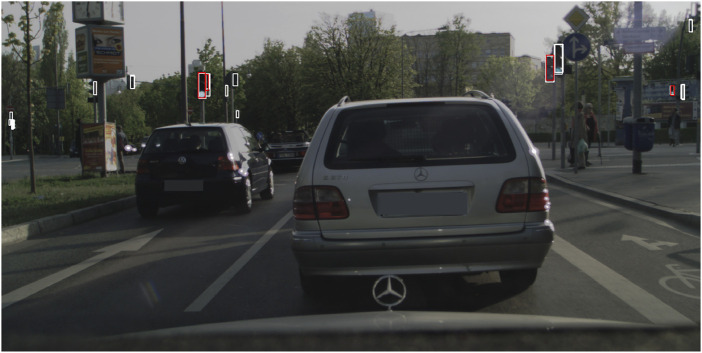
Sample image from the test set of DTLD. In white the detection of Faster R-CNN-R50-FPN, and red the labels annotated in the DTLD. It is possible to notice how the model can detect lights which are correct, but not annotated in the dataset’s ground truth.

After checking predictions on the sample images, we have noticed that Faster R-CNN-R50-FPN detects many traffic lights correctly that are not annotated or labeled as occluded in the DTLD, as can be seen in Figure 3. Especially for the same confidence threshold with other models, the false positives increase and disrupt the model’s results. However, for the HD map creation, as many traffic lights as possible should be mapped accurately. Thus, we have used the predictions of this model to track and map the traffic lights. Still, it is important to consider this when the results of Table 3 are considered. Indeed the real accuracy of this model should be higher than the one presented. For some classes, e.g., left and right directed, the Recall values of Table 3 are considerably low for some model. This is mostly due to the heavy unbalancing of the dataset. This issue cloud be solved with data augmentation techniques and proper tuning and balancing of the dataset. From our test this would slightly effect the other classes, which might be less accurate. Since the overall task is lights mapping we preferred an accurate model in the front direction because the survey vehicle is expected to navigate the area from multiple points of view, and it will always acquire lights images from the best position. The limited number of detection from other points of view are still useful to gather information and better define the lights position in the map.

**FIGURE 4:**
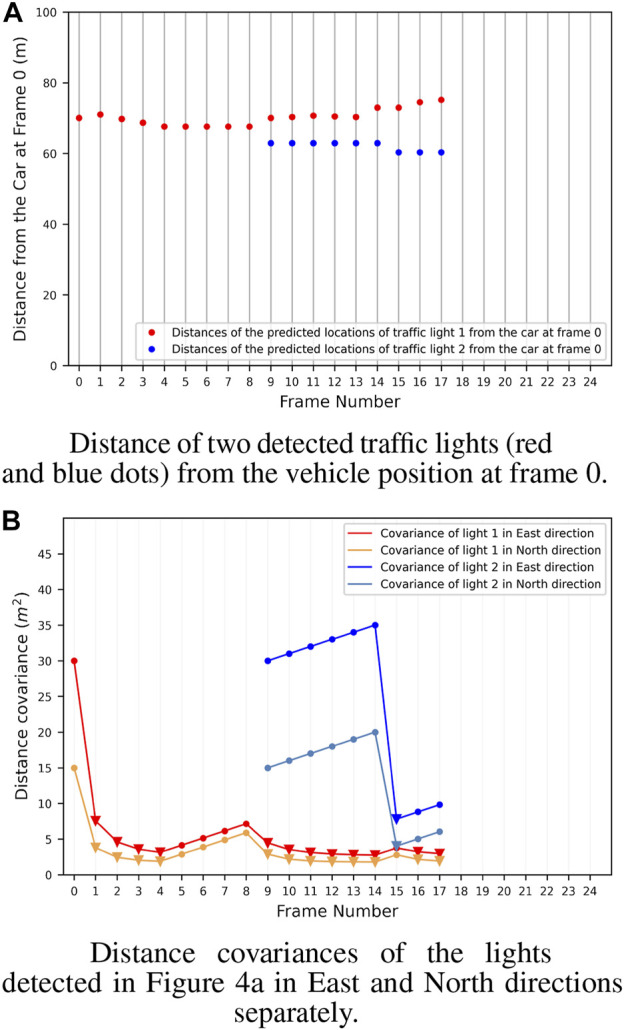
Distance **(A)** and Covariance **(B)** of two detected traffic lights in a sequence of 18 frames from the DTLD. It is interesting to notice how the covariance rapidly decrease when a new detection is performed. In particular the red light is detected many times and the covariance always maintain low values. Differently, the blue one is detected only at frame 9 and 15, therefore the covariance has only one drop when the new detection is performed.

**FIGURE 5:**
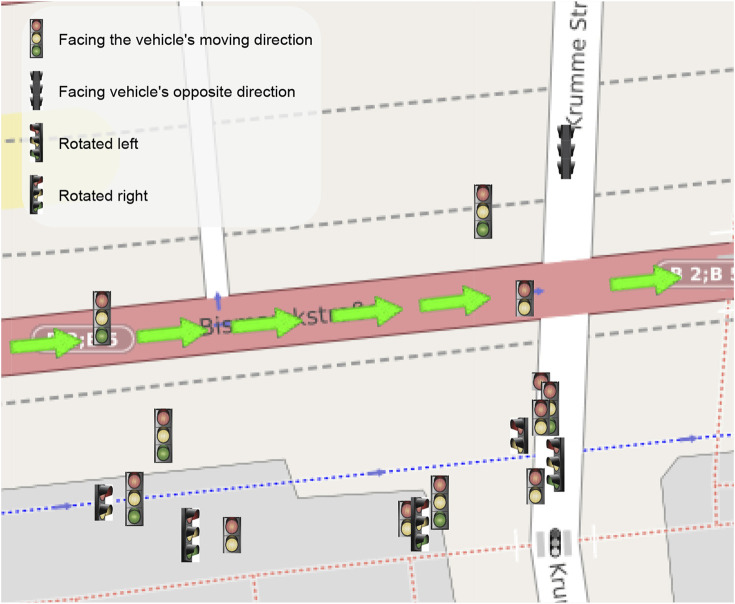
Map obtained from the pipeline on a sequence in the DTLD test set acquired on a complex junction in Berlin center (the green arrows represent the car trajectory). Close markers represent multiple lights mounted on the same pole, such as pedestrians, cyclists, buses, or tram lights. A slight offset has been applied to excessive close light for visualization purposes. The represented area is a small section of the whole 4 lanes crossroad.

**FIGURE 6:**
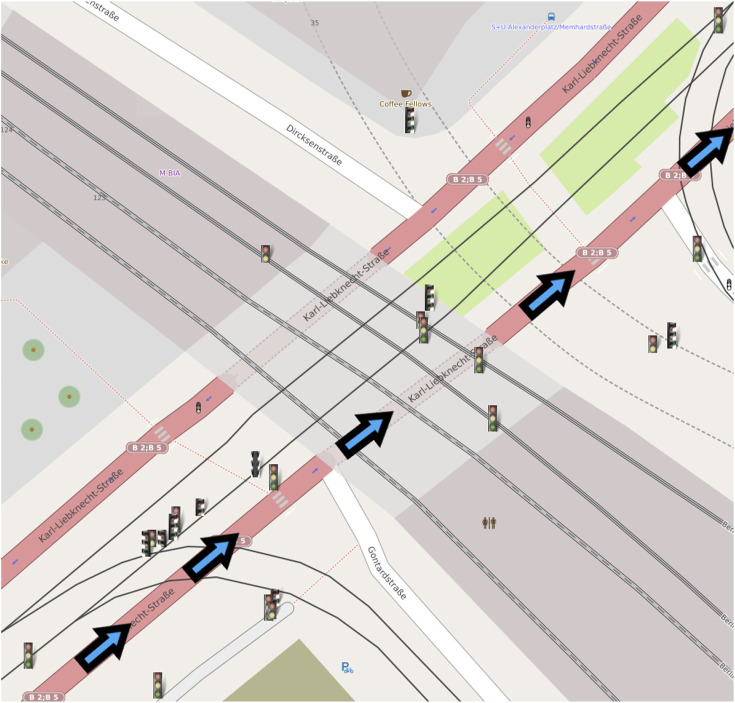
Second map with a lower resolution, obtained from a sequence of the DTLD test set.

Finally, to evaluate the results of the complete map creation process, in literature, there are metrics for multi-object tracking performance evaluation. However, the dataset does not provide a ‘real’ ground-truth for the traffic lights, but only the 2D bounding box on the image plane. This does not allow us to use these metrics, which would require the accurate GPS position of the lights. To qualitatively evaluate the performance of the tracking process, we have then plotted the position covariances of two sample traffic lights detected and tracked. The results of this process are shown in Figure 4. It is possible to appreciate how consecutive detections improve tracking accuracy in the plot. At the same time, position covariance gets larger with the skipped frames. In particular, Figure 4A shows the distance between two traffic lights from the vehicle. From the graph, it is possible to notice how the distance is always around the initial value, but get always adjusted at each frame using new detections. In Figure 4B the covariances of the position along the two axes for the lights are shown. For light one, it is possible to appreciate how the covariance of the position drops after the firsts detections. Then, the light is not detected from frame five to eight (circle points), and the covariance grows up, while when a new detection arrives (triangle points), the covariance drops again. This shows that the filter is working properly and integrates the new measures to refine the predicted light’s position. The final output of the system is then shown in Figure 5; Figure 6 where the whole traffic lights map is reconstructed on a test sequence of the DTLD dataset. It is possible to notice how the lights are correctly orientated, in a coherent way to the vehicle moving direction (green and blue arrows). The scenario represent a multi lines crossroads, therefore traffic lights are at different position on the *y* axis. Moreover, superimposed pictograms are due to detection of multiple lights mounted on the same pale with different orientation.

## 5 Conclusion

This work presents a complete pipeline for traffic lights detection and mapping for HD-map creation. To this end, we compared multiple deep-learning models, both for single-label and multi-label tasks. Unlike many traffic lights detectors in this work, we did not focus on state detection but all the other light features required by the HD-map creation process (i.e., shape, orientation, pictogram). Of particular interest is the ability of some models (i.e., Faster R-CNN-R50-FPN) to correctly detect lights at a distance, which are not present in the original dataset ground-truth. This makes it difficult to evaluate the network performance with standard metrics but simplifies the map creation process.

The light detector has been then integrated into a pipeline for map creation which combines the depth information computed from stereovision and the GPS position of the vehicle to create an accurate map of the lights. Next, all lights and vehicle positions are filtered using Kalman filtering to smooth the detected position, which is affected by GPS and stereo matching noise. The output is an accurate map with the position and features of all traffic lights seen by the vehicle, as shown in Figure 5 and Figure 6.

## Data Availability

Publicly available datasets were analyzed in this study. This data can be found here: https://www.uni-ulm.de/in/iui-drive-u/projekte/driveu-traffic-light-dataset/.
